# Cognitive flexibility is associated with sickness resilience

**DOI:** 10.3389/fpsyg.2024.1253152

**Published:** 2024-04-30

**Authors:** T. Vestberg, A. V. Lebedev, H. B. Jacobsen, M. Lekander, T. Sparding, M. Landén, L. Maurex, M. Ingvar, P. Petrovic

**Affiliations:** ^1^Department of Clinical Neuroscience, Karolinska Institutet, Stockholm, Sweden; ^2^Department of Psychology, University of Oslo, Oslo, Norway; ^3^Department of Psychology, Stress Research Institute, Stockholm University, Stockholm, Sweden; ^4^Department of Psychiatry and Neurochemistry, Institute of Neuroscience and Physiology, University of Gothenburg, Gothenburg, Sweden; ^5^Department of Medical Epidemiology and Biostatistics, Karolinska Institutet, Stockholm, Sweden

**Keywords:** executive functions, cognitive flexibility, design fluency, verbal fluency, sick leave, resilience, psychological flexibility

## Abstract

Psychological constructs related to health outcomes and well-being, such as metacognitive beliefs, have been linked to executive functions in general, and cognitive flexibility more specifically. However, such effects have previously only been discussed on a theoretical level and behavioral flexibility has most often been measured through self-report, only approximating information processing capacities. Objectively measured executive functions may be a more potent predictor of health outcomes. We set out to test whether cognitive flexibility capacity was associated with sick leave in a medium sized company. We included 111 subjects of widely different occupations and assessed their executive functions using Delis-Kaplan Executive Function System test battery (D-KEFS). To assess cognitive flexibility capacity, we included Design Fluency (DF) and Verbal Fluency (VF) and computed these into an index of cognitive flexibility (DFVF). Detailed information on sick leave for the last 5 years was gathered from the company. Our results showed that there was a significant negative correlation between DFVF and sick leave [r_s_(109) = −0.23, *p* = 0.015] in the full group as well as in the group that had at least 1 day of sick leave [r_s_(72) = **−**0.25, *p* = 0.03]. The results withstood adjustment for sex, age, occupation, and several core executive functions as well as autistic and ADHD-traits. The results remained for separate analyses using DF or VF. Our main findings were conceptually replicated in a group of bipolar disorder patients. This study shows that objectively measured capacity of cognitive flexibility is associated with key health outcomes such as sick leave.

## Introduction

The relation between cognition, behavior and health has received increasing attention. Several psychological constructs such as metacognitive beliefs (Kraft et al., 2017), psychological flexibility (Biglan et al., 2008; Kashdan and Rottenberg, 2010; Whiting et al., 2017; Gentili et al., 2019), resilience (Genet and Siemer, 2011; Kalisch et al., 2015; Hildebrandt et al., 2016), and locus of control (Declerck et al., 2006) are related to health and well-being. It has been suggested that executive functions (EF) play a critical role in these constructs (Declerck et al., 2006; Kashdan and Rottenberg, 2010; Kraft et al., 2017; Whiting et al., 2017). In line with this idea, EF have previously shown to be closely associated with health outcomes such as sick leave in patients (Biederman et al., 2006; Halleland et al., 2019; Drakopoulos et al., 2020). However, it is not known whether objectively measured EF relate to such health outcomes also in normally working individuals. The main goal of the present study was to investigate this relationship.

EF is an umbrella term describing cognitive processes that regulate thoughts and actions especially in non-routine situations (Diamond, 2013; Goldstein and Naglieri, 2014; Friedman and Miyake, 2017). The capacity of top-down regulation is distributed normally among a general population, and neuropsychiatric disorders like ADHD and autism are associated with low EF capacity in this distribution (Petrovic and Castellanos, 2016; Bayard et al., 2020). Cognitive flexibility (sometimes also denoted cognitive shifting, mental flexibility or set shifting) is an executive function that refers to the ability to switch mentally between tasks and rules, as well as shifting back and forth between multiple tasks, operations, mental sets, or strategies (Davidson et al., 2006; Diamond, 2013; Friedman and Miyake, 2017). It is associated with the ability to adjust to changed demands and is the opposite of rigidity (Diamond, 2013). Notably, cognitive flexibility has been suggested as a central component in several of the psychological constructs related to health discussed above, often as an aspect of more broad behavioral flexibility that is often rated (Genet and Siemer, 2011; Kraft et al., 2017; Whiting et al., 2017).

It has also been suggested that EF in general, and cognitive flexibility specifically, are central for successful behavior in diverse situations, speaking for its generic importance for individuals across demands. For example, we have previously shown that elite soccer players have better EF capacity, especially cognitive flexibility, than semi-elite players and population norm (Vestberg et al., 2012, 2017, 2020a). Along a similar line it has been suggested that cognitive flexibility is related to successful behavior associated with elite police forces tasks (Vestberg et al., 2020b), transformational leadership in management (Ramchandran et al., 2016) and driving performance (Ghawami et al., 2022).

In the present study, we aimed to move away from psychological constructs and rated behavioral flexibility, and instead test the hypothesis of whether an objective measure of EF capacity, with a focus on cognitive flexibility, is associated with sick leave in a normally working population. To test our assumption, we assessed EF in different occupational positions in a mid-size Swedish company that included strategic managers, operational managers, sellers and forklift operators. Thereby, we could also adjust for differences in socioeconomic status. We hypothesized that higher capacity of EF related to cognitive flexibility, would correlate negatively with reported sick leave over 5 years. Subsequently, we studied a group of bipolar patients to test whether our findings could be conceptually replicated in an unrelated setting.

## Materials and methods

### Participants

The study was performed in collaboration with a medium sized company in the wood products industry (Derome). A total of 111 co-workers between 22 and 67 years (mean age = 44.6, SD = 10.8; 94 males) participated (Supplementary Figure S1). Derome’s HR department offered employees to participate in the study. The participants represented a cross-section of sex, age and geographic location. Strategic managers, operating managers, sellers and forklift operators were included, mirroring different categories of employees in the company. Apart from these criteria, the employees were asked at random whether they would like to participate. Some exceptions were made concerning the location. If two employees were considered equivalent, the employee was chosen that was stationed closest to the places where the assessment was performed. The final sample of subjects consisted of 27 forklift operators, 25 sellers, 27 strategic managers and 32 operative managers (Table 1). None of the participants were on sick leave at the time of testing.

**Table 1:** Descriptive table of the included subjects and the occupation subgroups.

Occupation	Age	n (M/F)	Sick leave	DF	VF	DFVF
All subjects	44.6 (10.8)	111 (94/17)	4.6 (11.4)	11.7 (2.2)	12.1 (3.0)	11.5 (2.3)
Forklift operators	43.3 (14.5)	27 (24/3)	9.0 (13.5)	10.9 (2.0)	10.3 (2.8)	10.2 (2.1)
Operative managers	45.8 (8.5)	32 (30/2)	1.9 (2.1)	12.1 (1.9)	12.0 (2.5)	11.3 (1.8)
Sellers	42.4 (11.4)	25 (18/7)	5.8 (17.8)	12.0 (2.4)	12.6 (2.8)	11.9 (2.2)
Strategic managers	46.6 (8.4)	27 (22/5)	2.3 (6.0)	12.0 (2.2)	13.4 (3.3)	12.5 (2.7)

Age is given in years and sick leave in days per year. The results of the main tests of cognitive flexibility, including verbal fluency (VF), design fluency (DF) and the index consisting of both (DFVF), is given in average normalized scores. VF includes VF1 to 3 and DF includes of DF1 to 3. 10 is considered as the norm and 3 points on the scale equals one standard deviation. Standard deviation is shown in brackets.

### Ethics statement

The study was approved by the local ethical committee in Stockholm (2018/2574–31/1; 2019–00223) and the authors assert that all procedures contributing to this work comply with the ethical standards of the relevant national and institutional committees on human experimentation and with the Helsinki Declaration of 1975, as revised in 2008. All subjects were given verbal and written information on the study and gave their verbal and written informed consent to participate.

### Cognitive tests

We tested the capacity for cognitive and verbal flexibility using design fluency (DF) and verbal fluency test (VF) from the neuropsychological test instrument Delis-Kaplan Executive Function System test battery (D-KEFS) for our main cognitive assessments (Delis et al., 2001)—see Supplementary material. In order study a general flexibility component, we constructed an index of DF and VF denoted DFVF.

Design fluency and verbal fluency tests involve several EF-components, but are mainly considered to test for cognitive flexibility (Diamond, 2013). Latent structure analyses have shown that both tests include factors (named fluency and shifting) that are relatively independent from other EF component such as inhibition, processing speed and fluid reasoning (Karr et al., 2019; Furey et al., 2024). While VF1-2 and DF1-2 are considered to have a larger fluency component, VF3 and DF3 are considered to have a larger switching component (see further Supplementary Methods). Note, that also fluency includes flexibility (Diamond, 2013). The D-KEFS VF and DF tests also suggest ecological validity in behavior where behavioral flexibility is a key aspect (Vestberg et al., 2012, 2020a; Ghawami et al., 2022), with either larger effect than other executive functions or a remaining relation to the outcome when adjusted for core executive functions. The reliability of the D-KEFS tests is discussed in the Supplementary Methods.

We also used other tests of EF and low-level cognitive processing including the Stroop-task Color word interference (CWI) from (D-KEFS) and test for response time, simple attention, short-term memory and working memory and learning from CogStateSports (CS; Collie et al., 2003; Straume-Naesheim et al., 2005; see further Supplementary material). While the results from DF and VF were considered as higher order executive functions (HEF) due to the complexity of information processing, the other tests of EF were classified as measuring core executive functions (CEF) due to more limited EF components (Vestberg et al., 2012, 2017, 2020a) and primary used for adjustment of the main analyses and for exploratory reasons. These test were used for adjustments in our multiple regression analyses of various EF and low level cognitive processes that had less of a flexibility component than DF and VF but still are a part of the fluency tests. Thereby, our analyses would be more specific for cognitive flexibility.

### Procedure

The subjects went through the assessment at their working place. The assessments were made from March to June 2019. The subjects were tested in a standardized process with six test-leaders, trained and overseen by a licensed psychologist for this particularly assessment. There were no significant differences of the test results between the test-leaders.

### Primary outcome

In accordance with our hypotheses (see Supplementary material) our primary outcome was a combined measurement (mean of scaled scores) of DF1 (Filled dots), DF2 (unfilled dots), and DF3 (switching) together with VF1 (Letter), VF2 (Categories), and VF3 (Switching) to capture the creative abilities of generating solutions, rely on a creative ability paired with cognitive flexibility on both a visuo-spatial and verbal HEF level. By using a composite score (average normalized score of DF1-3 and VF1-3), we also reduced the noise of temporary test failures of the subjects. This primary index is referred to as DFVF (Design Fluency/Verbal fluency).

### Data on occupation, employment and sick leave

The company sampled sick leave data for all their employees. With permission from the test subjects, we collected information from the company that included occupation, months of employment and days of sick leave they have had 5 years back counted from the first of September 2019 (Mean = 50.6; Median = 60; SD = 15.8; Min = 3; Max = 60). The days of sick leave were then divided by how many months they have been employed (denoted as Days of Sick Leave) when used in our analyses. Occupation was assessed in order to adjust for difference in socio-economic measurements and different job-specific requirements and environment that may relate to both EF-capacity and sick leave.

### Psychiatric traits

We measured autistic and ADHD traits using Autism Spectrum Quotient (AQ; Baron-Cohen et al., 2001) and Adult ADHD Self-Report Scales (ASRS; Kessler et al., 2005), as these psychiatric traits are widely distributed in the population (Das et al., 2012; Ruzich et al., 2015) and known to affect executive function (Baron-Cohen et al., 2001; Willcutt et al., 2005; Crosbie et al., 2013; Floros et al., 2021; Seng et al., 2021), in order to perform sensitivity analyses and test whether our findings were not driven by these traits. For ASRS, each questions was scored from 0 to 4 and questions were divided into those assessing hyperactivity and those assessing inattention.

### Conceptual replication

In order to test whether we could conceptually reproduce our main findings we analyzed a data set with bipolar disorder patients from the St. Göran Bipolar Project (Drakopoulos et al., 2020). In that study 122 bipolar 1 and 2 patients were stratified into an active (*n* = 86) and an inactive group (*n* = 34) based on the number of hours per week working or studying. The results showed that executive functioning was a more powerful predictor of occupational status than IQ and other clinical factors, including illness severity in this patient group. Detailed description of the patient population is given elsewhere (Drakopoulos et al., 2020). This particular dataset was used for the analyses since it included the same tests of cognitive flexibility as in the present study (DF1-3 and VF1-3) and had data on sick leave (the patients rated number of sick days the last year). Patients that did not work or study were counted as having 100% sick leave. In the present study our conceptual replication aimed to assess whether there was an association between the degree of cognitive flexibility capacity using the same index as for the main analysis, i.e., DFVF, and sick leave. We first analyzed the full group and then only patients that were classified as active. As we analyzed the data only for replication purposes one-sided *p*-values were used.

### Statistical analysis

Our primary objective was to investigate the association between cognitive flexibility and higher executive functions (HEF), as quantified by the DFVF index, and the number of sick leave days within our study population. This detailed hypotheses are stated in Supplementary materials. Given skewed distribution of the sick leave data we employed Spearman’s Rho, a non-parametric correlation test, applying a two-tailed approach.

To avoid influence of individuals with no sick leave on the potential association between cognitive flexibility and sick leave, we conducted a subset analysis. This analysis included only participants who reported at least 1 day of sick leave, addressing our secondary hypothesis (see Supplementary materials for details). To account for potential confounders, we logarithmically transformed (log10) the sick leave data to approximate a normal distribution, as depicted in Supplementary Figure S2. For analytical transparency, initial evaluations on this transformed dataset included both non-parametric (Spearman’s Rho) and parametric (Pearson correlation) methods. Additionally, for exploratory purposes, we conducted a parametric analysis on each subtest of DF and VF (DF1, DF2, DF3, VF1, VF2, VF3) against the logarithmically transformed sick leave data. These results are detailed in the Supplementary materials.

Further analyses were conducted on participants with at least 1 day of sick leave using multiple linear regression, with stepwise adjustments for potential confounders. Here, Days of Sick Leave served as the dependent variable, while DFVF and other covariates were treated as independent variables. As the residuals were normally distributed we did not log-transformed the data. We evaluated the primary effect (DFVF ~ Sick leave) in a basic model (Model 1) and in successive models incorporating additional adjustments for demographic factors (Model 2: Sex, Age, Occupation), low-level cognitive functions and CEF (Model 3: Processing Speed, Attention, Working Memory, Learning, Inhibition), and psychiatric traits (Model 4: Autism and ADHD traits). “Occupation” categories included forklift operators, operative managers, sellers, and strategic managers. All low-level cognitive functions and CEF adjustments, except for Inhibition assessed through a Stroop task, were determined using a computerized test battery (details in Supplementary materials). Autism and ADHD traits were evaluated using the AQ (Baron-Cohen et al., 2001) and ASRS (Kessler et al., 2005) scales, respectively. To address potential issues of causality, reversed models (Sick leave ~ DFVF) are also reported in the Supplementary materials. Exploratory analyses of DF (average of DF1-3) and VF (average of VF1-3) as separate predictors were performed and are documented in the Supplements.

We performed also a separate multiple linear regression analyses to test whether there were any interaction between DF and Occupation. Days of Sick Leave served as the dependent variable, while DFVF and Occupation served as independent variables, now also introducing a interaction term in the model.

Additionally, we conducted a *post-hoc* analysis using Tukey’s Honestly Significant Difference (HSD) test to investigate differences in sick leave across various employee categories.

For the statistical procedures, we utilized IBM SPSS Statistics 25 for correlation analyses and Tukey’s HSD test. The Shapiro–Wilk test was employed to assess normality of distributions, and Levene’s test was used to verify the homogeneity of variances between groups. The multiple linear regression analyses were performed using the R language for statistical computing (version 4.4.2), within the RStudio environment (version 2023.06.1).

In our conceptual replication using the St. Göran Bipolar Project cohort (Drakopoulos et al., 2020), which included patients assessed with DF1-3, VF1-3, and reported sick leave days, our analysis strategy first encompassed all patients, including those not employed in the past year. Subsequent analyses focused on patients who were either working or studying. We hypothesized a negative correlation between DF and VF capacities, as measured by the DFVF index, and the number of sick days, mirroring our primary study findings. Due to the approximate nature of the sick leave data in this cohort and its non-normal distribution, non-parametric correlation tests were employed. Given the directional nature of our hypothesis, the reported *p*-values are one-sided.

## Results

### Descriptive data

Descriptive data regarding age, sex, occupation, sick leave, DF, VF and DFVF results for the included subjects is given in Table 1. There were no significant differences between the employment categories in terms of age or sex evaluated by analysis of variance and Chi-squared test, respectively (age: *F* = 1.493, df = 111, *p* = 0.224; sex: Xsq = 5.3478, df = 3, *p*-value = 0.148). Descriptive data on all variables used in the statistical analyses are presented in Supplementary Table S1. The main assumptions for the multivariate regression analysis were met (including normally distributed residuals). Inter-correlational table for the different measures used in the study are show in Supplementary Figure S3.

### Sick leave

For the whole group (n = 111) the mean of sick leave days the last 5 years was 0.38 days per month. Median sick leave was 0.083 days per month (Min = 0; Max = 7.5, SD = 0.95). While 74 of the included subjects had at least 1 day of sick leave during the last 5 years (Mean = 0.56 days per month; Median = 0.19; Min = 0.02; Max = 7.50, SD = 1.12), 37 of the participants had no days of sick leave. Due to the skewed distribution a log10 calculation was applied to the sick leave data for the included subjects who had at least 1 day of sick leave during the last 5 years for the correlation analyses (Supplementary Figure S2).

### Relation between DFVF and sick leave in the full group

We first performed an analysis that included all subjects studied where we had data on both sick leave and DFVF (*n* = 111). The non-parametric correlation analysis showed a significant negative correlation between DFVF and Days of Sick Leave [Spearman’s rho r_s_(*n* = 111) = −0.23, *p* = 0.015]. Two subjects displayed extreme values of sick leave. Repeating the analyses with these subjects removed rendered a similar result [Spearman’s rho r_s_(*n* = 109) = −0.19, *p* = 0.049].

### Relation between DFVF and Days of Sick Leave in subjects with sick leave

**Figure 1:**
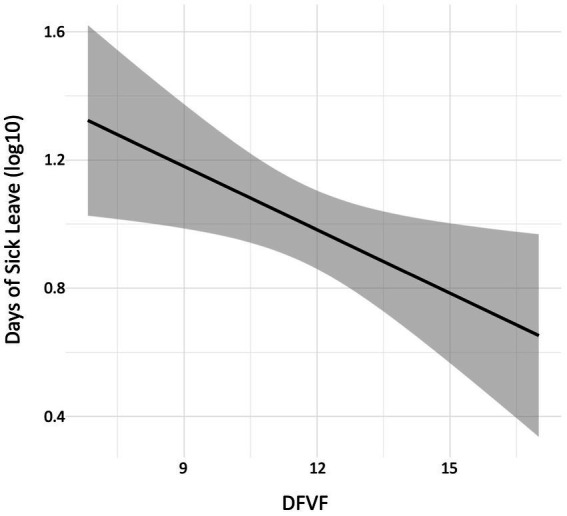
The correlation between the composite measure of design fluency and verbal fluency capacity (DFVF) and Days of Sick Leave (log10) for the subgroup who displayed at least one sick leave day during the observed period. Bands in grey color represent the 95% confidence intervals.

To investigate the quantitative relation between degree of sick leave and cognitive flexibility, we performed an analysis of the group that had at least 1 day of sick leave the last 5 years. We first tested whether the effects that we observed for the whole group remained for the group of subjects that had at least 1 day of sick leave the last 5 years (*n* = 74) using non-parametric correlational tests as above. The analysis showed a significant negative correlation between DFVF and degree of sick leave [Spearman’s rho r_s_(*n* = 74) = **−**0.25, *p* = 0.03]. After a log10 adjustment of the sick leave data (denoted as Sick leave log10) due to the skewed distribution, a normal distribution of the data was obtained (Supplementary Figure S2). A significant negative correlation between sick leave (Sick Leave log10) and DFVF remained in this analysis (Pearson r(72) = **−**0.27, *p* = 0.02) as illustrated in Figure 1.

### Relation between DFVF and Days of Sick Leave adjusting for other parameters

A multiple linear regression analysis was then performed to study the relation between Day of Sick leave (dependent variable) and DFVF (Model 1) and when adjusting stepwise for demographics and socioeconomic factors (sex, age, occupation) in Model 2, low level cognitive factors and CEF (processing speed, attention, working memory, learning and inhibition) in Model 3, as well as ADHD and autism traits in Model 4 (using ASRS and AQ questionnaires). In the analysis, the relation between sick leave and DFVF remained significant in all models (Table 2), suggesting that lower sick leave was related to better results on DFVF also after the adjustments. We also found a significant relation between Days of Sick Leave and occupation (Model 3 and 4) and inhibition (Model 4) in this analysis (Table 2). The other independent variables did not show any significant effect in this analysis. As it is not certain whether low cognitive flexibility capacity causes increased sick leave or whether clinical conditions associated with sick leave causes low cognitive flexibility we also show the same analyses in which DFVF is the dependent variable. A significant relation remained in all models (Supplementary Table S2).

**Table 2:** Multiple linear regression analysis in which we analyzed the relation between Days of Sick Leave (dependent factor) and DFVF with four models adjusting stepwise for potential cofounders.

	Model 1			Model 2			Model 3			Model 4		
Variable	Beta	95% CI1	*p* value	Beta	95% CI1	*p* value	Beta	95% CI1	*p* value	Beta	95% CI1	*p* value
DFVF	−4.7	−7.8, −1.7	0.003	−4.4	−7.5, −1.2	0.009	−6.0	−9.6, −2.3	0.002	−6.4	−10, −2.8	0.001
Age				0.49	−0.13, 1.1	0.12	0.48	−0.17, 1.1	0.2	0.66	−0.02, 1.3	0.061
Sex				−8.6	−27, 9.9	0.4	−13	−33, 6.6	0.2	−17	−38, 2.9	0.10
Occupation				6.0	−0.34, 12	0.068	7.5	1.0, 14	0.027	9.1	2.6, 16	0.008
Processing Speed							0.00	−1.0, 1.0	>0.9	0.10	−0.92, 1.1	0.8
Attention							0.12	−0.48, 0.73	0.7	0.12	−0.48, 0.71	0.7
Working Memory							0.28	−0.90, 1.5	0.6	0.20	−0.96, 1.4	0.7
Learning							0.02	−1.1, 1.2	>0.9	0.18	−0.95, 1.3	0.8
Inhibition							3.5	−0.02, 7.1	0.056	3.7	0.21, 7.2	0.042
AQ										−1.2	−2.6, 0.22	0.10
ASRS—Attention										1.1	−0.75, 2.9	0.2
ASRS—Hyperactivity										0.78	−0.73, 2.3	0.3

Model 1 is without adjustments. In subsequent models adjustments for demographics and occupation (Model 2), cognitive functions (Model 3) and psychiatric traits (Model 4) have been added stepwise.

### Testing for interaction between DFVF and occupation

In a separate ANOCOVA where Days of Sick Leave served as the dependent variable, while DFVF and Occupation served as independent variables and an interaction term was introduced, the relation between Days of Sick Leave and Occupation as well as between Days of Sick Leave and DFVF remained significant (*F* = 5.52, *p* = 0.022 and *F* = 6.32, *p* = 0.014, respectively) while no interaction was observed (*F* = 1.25, *p* = 0.27).

### Post-hoc analyses of the result of DFVF in the different working groups

A post-hoc analysis on the full group (*n* = 111), using Tukey’s HSD indicated that the scores of DFVF were significantly higher for the strategic managers (*p* = 0.002), operating managers (*p* = 0.017), and sellers (*p* = 0.022) compared with forklift operators. Restricting the analyses to the group with at least 1 day of sick leave (*n* = 74), indicated that the scores of DFVF were significantly higher for the operating managers (*p* = 0.031) compared with forklift operators. Sellers (*p* = 0.062), and strategic managers (*p* = 0.14) did not display significantly higher results than forklift operators.

### Relations between Days of Sick Leave and DF and VF, respectively

To explore if the verbal and the visuo-spatial part of the fluency measurement are independently associated with sick leave, multiple linear regression analyses were performed in the same way as for DFVF but separately for DF (Design fluency) and VF (Verbal fluency) in the subjects that had at least 1 day of sick leave. These analyses showed that Days of Sick Leave was significantly related to DF and VF separately in all models (Supplementary Tables S3, S4).

Finally, for transparency we present how DF1-3 and VF1-3 independently related to sick leave in subjects who had at least 1 day of sick leave. In general, all subtests seemed to have a similar relation to sick leave (Supplementary Table S5).

### Results of conceptual replication

The bipolar patient group, in which we performed a conceptual replication analysis, showed a negative correlation between DFVF and sick leave. These effects were both shown in the full group of patients (*n* = 122; Correlation Coefficient: −0.252; *p* < 0.005), and also on a threshold level, when individuals with no work / study days for the full year were excluded (*n* = 95; Correlation Coefficient: −0.168; *p* = 0.0502).

## Discussion

The results of this study supported our main hypothesis, by showing that a composite score of cognitive flexibility incorporating both design fluency and verbal fluency (DFVF) correlated inversely with degree of sick leave in a mixed population of employees at a mid-size company. This effect was shown for the full group as well as for the subgroup that had at least 1 day of sick leave during the last 5 years. In the subgroup, the effect remained when adjusting for sex, age, and occupation as well as for several low-level cognitive measures like general response speed, simple attention, short-term memory, working memory and inhibition. By adjusting for these factors, it is unlikely that basic cognitive functions and other CEF could fully explain the results. This suggests that a combination of cognitive flexibility and fluency may be a main factor relating to the degree of sick leave. We conceptually replicated our main results in another data set consisting of a bipolar group (Drakopoulos et al., 2020).

The present result is of interest since sick leave is an important indicator of health and work ability. While influential psychological models such as metacognitive theory (Papageorgiou and Wells, 2009; Jacobsen et al., 2016, 2020; Kraft et al., 2017; Capobianco et al., 2018), psychological flexibility (Biglan et al., 2008; Kashdan and Rottenberg, 2010; Whiting et al., 2017; Gentili et al., 2019), resilience (Genet and Siemer, 2011; Kalisch et al., 2015; Hildebrandt et al., 2016) and locus of control (Declerck et al., 2006) imply that EF and cognitive flexibility may be an important aspect of a positive health outcome, this has not been specifically tested in a normally working population. In line with such a hypothesis, we show that capacity of cognitive flexibility measured with neuropsychological tests, is related to a central health outcome such as sick leave. Below we discuss the relation between these psychological concepts, EFs and health outcomes.

Of particular interest here, is that design and verbal fluency can be viewed not only as tests of flexibility but also tests of creativity within a set of rules. In order to be successful on the test, relevant rules must be selected, understood and actions planned. Moreover, the chosen strategies must be flexibly adjusted, and the utility of strategies constantly evaluated. All these functions are metacognitive in nature, making it tempting to speculate that the current association between fluency and work participation could be mediated by flexible metacognitive strategies and beliefs. Past research has shown how rigid and negative metacognitive beliefs affect duration of sick leave (Jacobsen et al., 2020). Such beliefs also have a negative effect on mental capacity (Wells and Matthews, 1996) and are associated with subjective cognitive deficiencies (Jacobsen et al., 2016). Moreover, negative metacognitive beliefs have been linked to objective neuropsychological impairments (Kraft et al., 2017), cognitive flexibility in particular. Thus, the association between cognitive flexibility and metacognitive capacity may go both ways.

A similar proposal may be suggested for the construct psychological flexibility. Namely, a reduced flexibility in mindsets and behavior is increasing the risk for psychopathology such as depression and anxiety but also clinical symptoms such as pain (Kashdan and Rottenberg, 2010). For example, inflexibility of thoughts will lead to rumination that involves stereotypical and perseverative thinking, which will increase the risk for depression. Notably, the results of our study are in line with studies indicating a negative relation between psychological flexibility and sick leave in chronic pain patients (Hara et al., 2018; Gentili et al., 2019). However, in those studies psychological flexibility was estimated from self-ratings, while the capacity of cognitive flexibility from a more objective performance measurement was not assessed.

We propose that the relation between cognitive flexibility capacity and lower sick leave is the same as in the psychological constructs discussed above. While such constructs relate more to a conceptual level, cognitive flexibility relates to the information processing level. Common reasons for sick leave are back- and neck pain, cardiovascular disorders, and mental disorders (Alexanderson et al., 2003; Lidwall, 2015). Our study did not differentiate between causes for sick leave. However, we suggest that both mental and somatic health may be influenced by HEF and cognitive flexibility capacity, and thereby work ability. A well-developed cognitive flexibility may work as a resilience factor and improve problem-solving, that, in turn, may impact on health relevant factors such as stress sensitivity, sleep, exercise, diet, immune function or pain processing (Solberg Nes et al., 2009; Berryman et al., 2014; Ortego et al., 2016; Marsland et al., 2017). Moreover, increased cognitive flexibility may lead to less rumination and fewer rigid metacognitive beliefs that are associated with development of depressive states. Similarly, cognitive control of demanding situations may influence the perceived control and thus buffer stress level and contribute to adaptation (Shields et al., 2017). In speculation, such control, and the ability to properly adapt behaviors to demands may contribute to perceived work ability and thereby a lower inclination for sick leave. In support of this proposition, several studies indicate that low job control, among other factors, is associated with higher risk for sickness absence (Indregard et al., 2017; Duchaine et al., 2020; Norberg et al., 2020) and predicts early return-to-work in sick listed employees (Indregard et al., 2017).

All the factors discussed above have a putative effect on both subjective and objective correlates of health, and thereby sick leave mediated by EF and cognitive flexibility. However, there are alternative explanations behind the current results. Both VF and DF also include other cognitive aspects than cognitive flexibility. Although DFVF was related to sick leave also when adjusting for other cognitive variables (e.g., general attention, processing speed, short term memory, working memory and inhibition), it cannot be excluded that other cognitive variables do not influence the results. Although closely associated to cognitive flexibility, cognitive fluency and creativity, DF and VF may involve additional EF processes not accounted for (Delis et al., 2001; Diamond, 2013; Karr et al., 2019). More specific motor, perceptual and language components may impact the test results (Suchy et al., 2010; Whiteside et al., 2016). It may for example be of importance how well-developed language a subject has for the VF result or how well-developed low-level perceptual abilities a subject has for the DF results. However, we show that both VF and DF contribute to this relation to a similar degree. Moreover, the complexity of the tests may also have influenced the results, as both VF and DF may be regarded as HEF tests involving many simultaneous EF and non-EF components increasing the general cognitive load on the information processing system. Finally, high EF are associated with better emotional regulation (Solberg Nes et al., 2009; Petrovic and Castellanos, 2016), including regulation of pain as well as anxiety and depression, and may explain our results independent of any relation to the discussed psychological models. In order to link our findings to the psychological models presented previously, the relation between tested cognitive flexibility capacity and measurements of behavioral flexibility in life needs to be investigated.

Although our results are novel for the normally working population they are in line with several studies on patients. For example, it has been shown that EF may predict absence from work following in-patient occupational rehabilitation (Johansen et al., 2021) as well as for individuals with ADHD (Biederman et al., 2006; Halleland et al., 2019). Another study compared remitted bipolar patients that were able to continue to be active (studying or working) with those who were inactive (e.g., unemployed, received early retirement benefit or being on long term sick leave; Drakopoulos et al., 2020). The group that still was active had significantly better IQ and EF capacity including cognitive flexibility, as compared to the inactive group. However, only EF significantly accounted for the variance in occupational status between the active and non-active patient group. These results complement those of our study, which showed a correlation between cognitive flexibility and sick leave in a normal working population. Apart from the difference in studying patients vs. normal population, our study showed that the results remained after adjusting for several core executive functions, while the bipolar patient study discussed above (Drakopoulos et al., 2020) suggested that the effect was not related to IQ-level.

To further test whether our findings could be conceptually replicated we performed a similar analysis on the group of bipolar patients discussed above from the St Göran project (Drakopoulos et al., 2020) as they had rated number of sick days the last year and performed both DF and VF tests. These analyses showed that DFVF correlated negatively with rated days of sick leave both when all patients were included, but also when patients that did not work or study at all were excluded (on a threshold significant level). Thus, it suggests that a close relationship between cognitive flexibility and sick days is present also in patient groups, including in those that still are active. The days of sick leave were rated by the patients in St Göran project, making this information somewhat less reliable in comparison to the main analyses or to studies using register-based information of sick leave. An advantage with our main analysis studying healthy subjects, as compared to registry-based studies, is that short-term sick leaves (obtained from the employer) were included. In comparison only sick leaves of 14 days or longer are most often used in registered based studies, e.g., studies based on Swedish Social Insurance Agency.

An alternative explanation of the results involves reversed causality. Reduced EF may be a consequence of previous or ongoing health problems, rather than the opposite. It is, for example, possible that previous depression is related to a deteriorating EF capacity. Likewise, although no subjects were on sick leave at time for assessment, it is possible that subjects with a high degree of sick leave experienced sub-clinical levels depression, anxiety, sleep problems or other health problems that might negatively impact higher order executive functions capacity when tested. Thus, the study cannot show that EF-capacity impact development in number of sick days. However, our results survived sensitivity analyses in which we adjusted for ADHD- and autistic traits that are widely spread in the general population (Das et al., 2012; Ruzich et al., 2015) and are known to affect executive functions (Baron-Cohen et al., 2001; Willcutt et al., 2005; Crosbie et al., 2013; Floros et al., 2021; Seng et al., 2021). Future research could possibly resolve the causality question by longitudinally following a subjects related to their clinical status, executive functions and sick leave.

Our study showed that managers had significantly higher HEF and lower amount of sick leave compared to forklift operators, that had lower HEF and higher amount of sick leave. A secondary consequence of well-developed HEF could be that managers are less vulnerable to stress and therefore have less sick leave (Öhman et al., 2007). However, it is unclear if the difference in sick leave between these groups is a consequence of cognitive capacity or difference in their working situation. Importantly, the negative correlation between sick leave and the DFVF remained even when adjusting for occupation and there was no significant interaction between occupation and DFVF in an analysis where Days of Sick Leave was used as an dependent factor. Thus, the main result seems not to be dependent on socioeconomic status.

There are several limitations in this study. Measures of IQ were not available. Another limitation is that we did not measure psychological flexibility and can only speculate about how it may relate to cognitive flexibility capacity.

On a more general level this study suggest that well-developed EF could have a positive impact in healthy and fully functioning subjects. It is aligned with previous studies showing a relation between EF and high performance in ball sports (Vestberg et al., 2012, 2017, 2020a), elite police officers (Vestberg et al., 2020b) and leadership positions (Ramchandran et al., 2016). The results also suggest a cognitive mechanism, i.e., cognitive flexibility, that may underlie a general flexible behavior emphasized in several psychological constructs and often rated subjectively. Further studies should better describe this possible link between flexible behavior in life and an objectively measured cognitive core capacity (Petrovic and Castellanos, 2016).

## Data availability statement

Raw data supporting the conclusions of this article that does not identify specific individuals will be made available by the authors, without undue reservation.

## Ethics statement

The studies involving humans were approved by Swedish Ethical Review Authority. The studies were conducted in accordance with the local legislation and institutional requirements. The participants provided their written informed consent to participate in this study.

## Author contributions

TV, PP, and MI designed the study. TV performed the testing. TV, AVL, and PP analyzed the data. All authors interpreted the results and wrote the manuscript. All authors contributed to the article and approved the submitted version.
