# Supplementary material for: Cognitive flexibility is associated with sickness resilience
Source: Front Psychol. 2024 Apr 30;15:1253152. doi: 10.3389/fpsyg.2024.1253152 (PMC11091381; doi:10.3389/fpsyg.2024.1253152)
Supplement: Supplementary file 1 [file Supplementary_Materials.pdf]

Online Supplementary material

Cognitive flexibility is associated with sickness resilience

T. Vestberg[1], A. V. Lebedev[1], H. B. Jacobsen[2], M. Lekander[1,3], T. Sparding[4], M. Landén[4,5], L. Maurex[1], M. Ingvar [1] and P. Petrovic[1]*

[1] Department of Clinical Neuroscience, Karolinska Institutet, Stockholm, Sweden

[2] Department of Psychology, University of Oslo, Oslo, Norway

[3] Stress Research Institute, Department of Psychology, Stockholm University, Sweden

[4] Department of Psychiatry and Neurochemistry, Institute of Neuroscience and Physiology, University of Gothenburg, Gothenburg, Sweden.

[5] Department of Medical Epidemiology and Biostatistics, Karolinska Institutet, Stockholm, Sweden

e-mail: predrag.petrovic@ki.se

Hypotheses

Main hypothesis: DFVF, an index representing higher order executive functions (HEF) that includes a high degree of cognitive flexibility, is associated with sick leave in the studied population.

Secondary hypotheses: DFVF is associated with sick leave in the subpopulation consisting only of individuals with at least on day of sick leave. The relation remains when adjusting for sex, age, working group, processing speed, and CEF (including simple attention, short-term memory, working memory and inhibition).

Exploratory analysis: Is DFVF different in relation to the different occupations? Are VF and DF separately associated with sick leave?

Tests used in the present study

D-KEFS

We used the neuropsychological test instrument The Delis-Kaplan Executive Function System test battery (D-KEFS) for our main cognitive assessments (Delis et al., 2001). D-KEFS is a test battery measuring different aspects of executive functions (EF). All tests in the battery are performance tests. Primary measurement is often time in seconds. Secondary measurement is often accuracy. D-KEFS is used in clinical assessments and there are well-described norms for the general population. A test-retest reliability analysis for the D-KEFS tests has been performed on the D-KEFS norm group (1750 individuals stratified on age, sex, ethnicity and education) in the US and showed a moderate to strong reliability (Delis et al., 2001;Delis et al.,

2004). D-KEFS tests show a normal distribution in healthy subjects (Delis et al., 2001;Karr et al., 2019) and the results relate to brain morphology (Abe et al., 2018;Mace et al., 2019) within networks involved in EF (Cieslik et al., 2015). Latent structure analyses have shown that D-KEFS tests include several EF-factors in which DF and VF tests often converge on the same factor (Miyake et al., 2000;Karr et al., 2019;Furey et al., 2024).

Subtests of D-KEFS

Design Fluency (DF), is a standardized test which measures on-line multi-processing such as planning, working memory, visual scanning, creativity, response inhibition, and cognitive flexibility (Delis et al., 2001;Homack et al., 2005;Suchy et al., 2010;Diamond, 2013) and thus simulates the executive chain of decision making that may be relevant for fast and accurate behavior. Especially, cognitive flexibility and the closely associated creativity components are emphasized in these tasks. It has also been argued that DF is a test of higher executive functions (HEF) since it incorporates several core executive functions (CEF) that are used in order to complete the task (Delis et al., 2001;Vestberg et al., 2017;Sakamoto et al., 2018). DF is a non-verbal psychomotor test in which the participant uses a pen to combine dots in a square with four lines. In Condition 1 (Design Fluency 1; DF1) the task is to find as many different combinations as possible of binding together filled dots under time pressure (60 seconds) and the participant is not allowed to use a solution twice. In Condition 2 (Design Fluency 2; DF2) unfilled dots have been added to the square, and the task is to combine them with lines as in Condition 1. The filled dots are still present but the participant is not allowed to use them in the task. In this condition the task raises the general level of difficulty due to more need from response inhibition (Delis et al., 2001). In Condition 3 (Design Fluency 3; DF3) both filled and unfilled dots are still present, and the task is to connect lines as above but also to constantly switch between a filled and an unfilled dot. Thereby, the task difficulty increases through the

raised demand on the subject's ability in cognitive flexibility (Delis et al., 2001). All scores are expressed as normalized values adjusted for age and sex.

Verbal fluency (VF) is a standardized task that, like DF, measures on-line multi-processing such as planning, working memory, creativity, response inhibition, and cognitive flexibility, but with a semantic output (Delis et al., 2001;Diamond, 2013). In Condition 1 (Verbal fluency 1; VF1), Letter Fluency, the task is to say as many words as possible on a given letter during 60 seconds without making any repeats and observing several rules and restrictions. In Condition 2 (Verbal fluency 2; VF2), Category Fluency, the task is to say as many words as possible from a given category during 60 seconds without repeating words. In Condition 3 (Verbal fluency 3; VF3), Category Switching, the task is to say as many words as possible in 60 seconds without repeats from two different categories and alternating between the categories after every reported word. All scores are expressed as normalized values adjusted for age and sex.

Additional tests that were used

Additional cognitive tests that were not part of the main hypothesis but used to adjust for cognitive low level processes and CEF in our models.

Color word interference (CWI) is a Stroop-test involving verbal inhibition (Delis et al., 2001;Diamond, 2013) from D-KEFS test battery (Delis et al., 2001). In test-condition 1 (CWI-1) the participant is instructed to say the printed color of the squares (green, blue and red), line by line, from the top to the bottom of a paper. In test-condition 2 (CWI-2) the participant is instructed to read the color words (green, blue and red) in black ink, line by line, from the top to the bottom of a paper. In test-condition 3 (CWI-3) all color words (green, blue and red) are either printed in the congruent or incongruent color and the subject is instructed to report the

printed color. The test captures response inhibition and represents the classical form of the Stroop task.

CogStateSport

CogStateSports (CS) is a non-verbal psychomotor test battery that measures basic attention, cognitive process speed, decision-making, speed and accuracy of short-term memory and encoding of working memory (Collie et al., 2003;Straume-Naesheim et al., 2005). It has high reliability in healthy adult subjects (Collie et al., 2003). The subjects are shown different playing cards on a computer screen and have to react as fast and correct as possible using different key responses. In the first test ("**Processing speed**"), measuring simple response time, the subject has to respond to any card that is displayed. In the second test ("**Attention**"), measuring simple attention, the subject has to respond whether the card is red or black. In a third test ("**Learning**") the subject has to respond if he or she has seen the displayed card any time earlier in the test sequences - a measure of more demanding working memory and learning. In the fourth test ("**Working memory**"), measuring the short-term memory, the subject has to decide if the previous card is the same as the card before (i.e. one-back memory-test).

Figure S1.

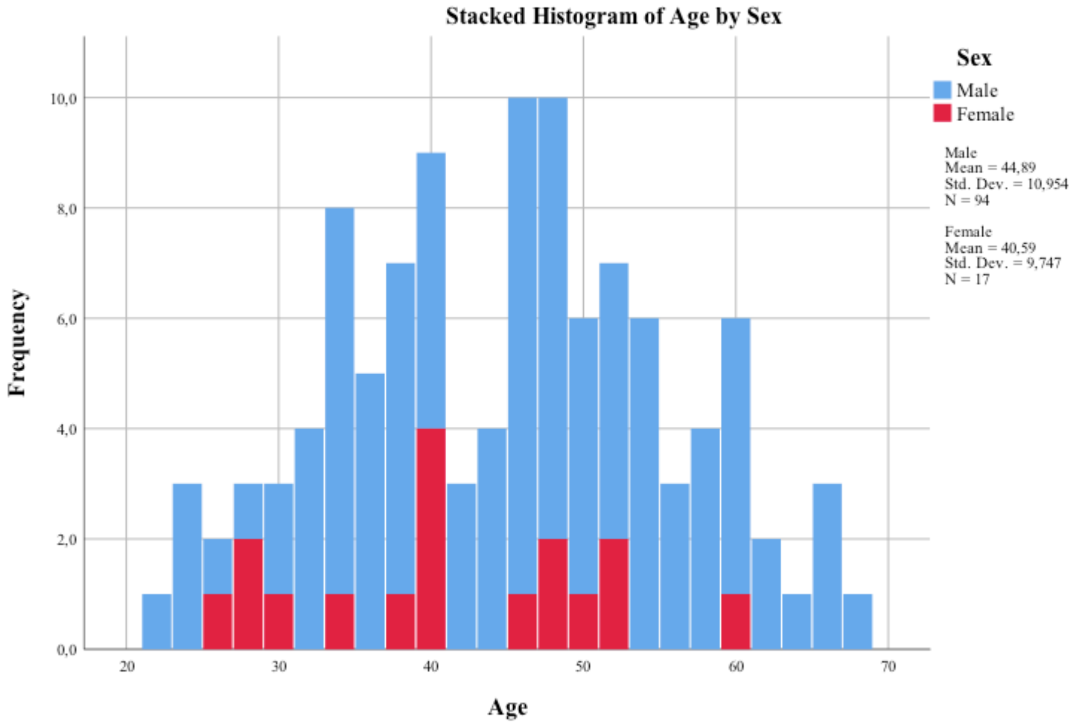

Figure S1. Age frequency of the test-group.

Figure S2.

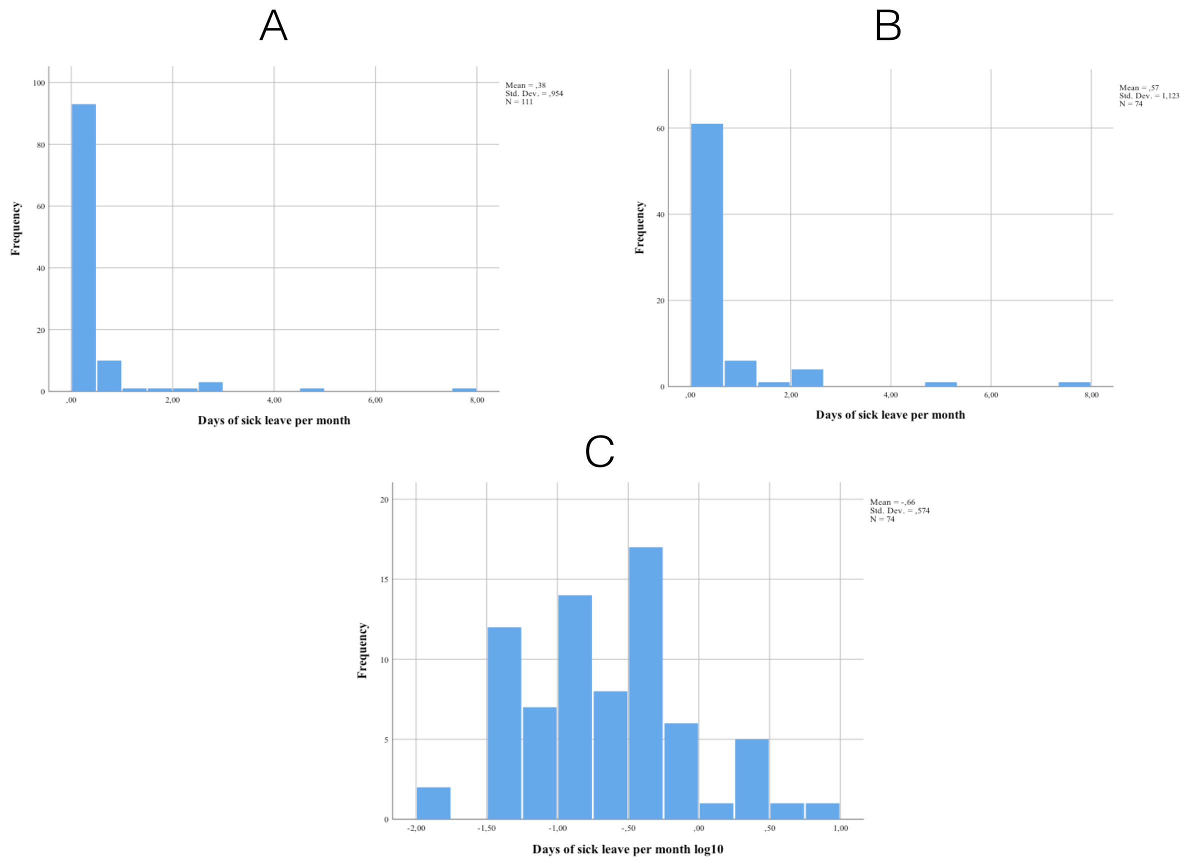

Figure S2. A) Histogram of the distribution of the sick leave the last 5 years (day per month) in A) the full group of subject (n=111) and B) the group that had at least one day of sick leave the last five years (n=74). C) Log10 of the sick leave the last 5 years (day per month) in the group that had at least one day of sick leave the last five years. This data was normally distributed (Shaprio-Wilk p=0.20).

Figure S3.

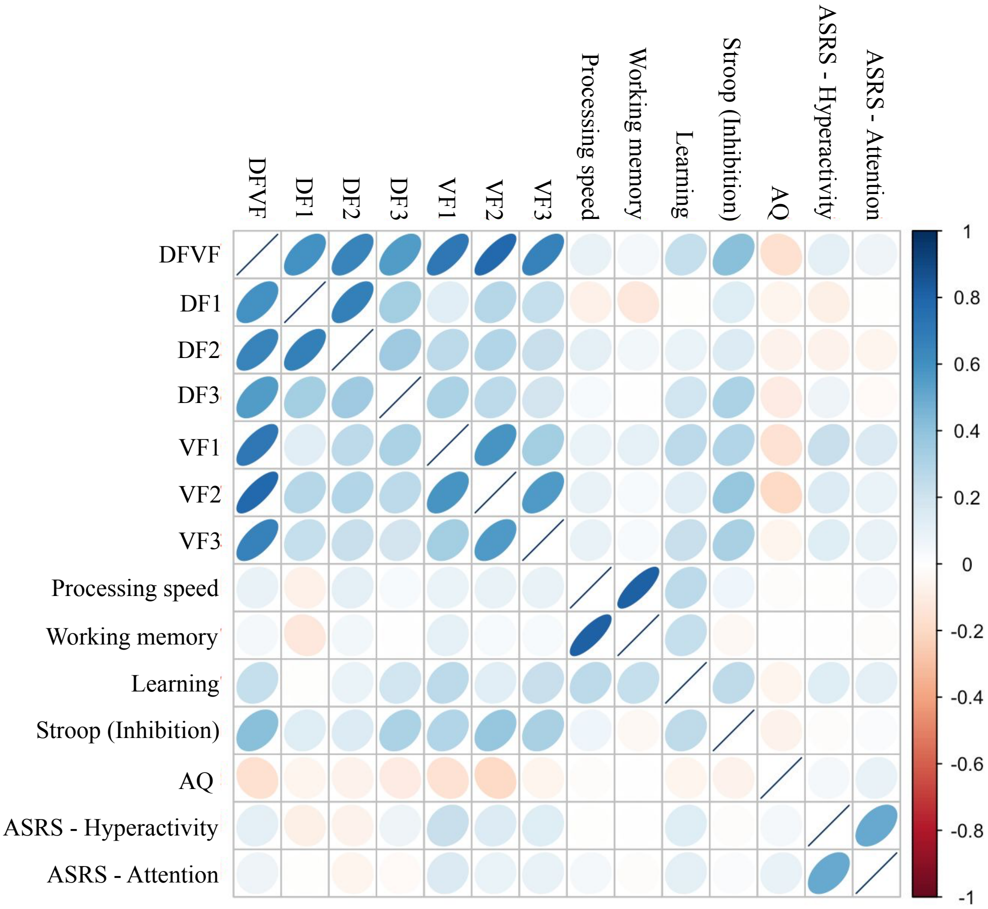

Figure S2. Inter-correltional table for the different measures used in the study.

Table S1.

Measure	Mean	SD	Skewness	Min	Max
Days of Sickleave	21.5	31.14	2.65	1	153
DFVF	11.73	2.21	0.074	6.83	17
DF1	11.59	2.56	0.61	8	19
DF2	11.34	2.55	0.15	6	19
Df3	11.73	2.81	-0.24	4	18
VF1	11.24	3.92	0.13	2	19
VF2	13.59	4.05	-0.34	5	19
VF3	10.76	3.48	0.22	4	18
Processing Speed	101.03	12.90	-6.60	0	114.4
Working Memory	94.62	11.43	-6.70	4.8	102.6
Learning	104.16	7.19	-0.021	85.7	126.3
Stroop (Inhibition)	10.74	2.26	-1.14	3	14
AQ	13.57	4.73	0.62	5	28
ASRS - Attention	12.58	4.21	0.032	3	22
ASRS - Hyperactivity	12.54	5.24	0.43	3	28

Table S1. Main results for the different measures used in the study. DF, VF and Stroop task (Color Word Interference) are D-KEFS-tests where 10 is the normlized average for the norm (Delis et al., 2001). 3 points equals one standard deviation in the norm. DFVF is an average of DF and VF. Processing Speed, Workning Memory and Learning, are CogStateSport-tests where 100 is the normlized average for the norm (Collie et al., 2003;Straume-Naesheim et al., 2005). AQ = Autism Spectrum Quotient (Baron-Cohen et al., 2001). ASRS = Adult ADHD Self-Report Scales (Kessler et al., 2005). The ASRS was divided into the hyperactivity and Attention part. In ASRS each questions was scored from 0 to 4.

Table S2.

	Model 1			Model 2			Model 3			Model 4		
Variable	Beta	95% CI1	p-value	Beta	95% CI1	p-value	Beta	95% CI1	p-value	Beta	95% CI1	p-value
Days of Sick Leave	-0.02	-0.04, -0.01	0.003	-0.02	-0.04, -0.01	0.009	-0.02	-0.04, -0.01	0.002	-0.03	-0.04, -0.01	0.001
Age				0.00	-0.04, 0.05	0.8	0.01	-0.03, 0.05	0.6	0.01	-0.03, 0.06	0.6
Sex				-1.2	-2.5, 0.04	0.063	-1.6	-2.8, -0.46	0.008	-2.0	-3.2, -0.76	0.002
Occupation				-0.35	-0.80, 0.10	0.13	-0.02	-0.44, 0.40	>0.9	0.04	-0.39, 0.48	0.8
Processing Speed							0.01	-0.05, 0.08	0.7	0.02	-0.05, 0.08	0.6
Attention							0.00	-0.04, 0.04	>0.9	0.00	-0.03, 0.04	0.8
Working Memory							-0.02	-0.09, 0.06	0.7	-0.02	-0.09, 0.05	0.6
Learning							0.07	0.00, 0.14	0.040	0.08	0.01, 0.15	0.033
Inhibition							0.35	0.14, 0.56	0.002	0.36	0.15, 0.57	0.001
AQ										-0.07	-0.16, 0.02	0.14
ASRS - Attention										0.09	-0.02, 0.21	0.11
ASRS - Hyperactivity										-0.02	-0.12, 0.07	0.6

Table S2. Linear regression analysis with four models adjusting for potential cofounders using DFVF as dependent variable to study its relation to Days of Sick Leave. Model 1 is without adjustments. In subsequent models adjustments for demographics and occupation (Model 2), cognitive functions (Model 3) and psychiatric traits (Model 4) have been added stepwise.

Table S3

	Model 1			Model 2			Model 3			Model 4		
**Variable**	Beta	95% CI1	p-value	Beta	95% CI1	p-value	Beta	95% CI1	p-value	Beta	95% CI1	p-value
DF	-4.5	-7.7, -1.3	0.007	-4.0	-7.2, -0.72	0.019	-4.5	-7.9, -1.0	0.014	-4.2	-7.7, -0.75	0.020
Age				0.42	-0.21, 1.1	0.2	0.38	-0.30, 1.1	0.3	0.55	-0.16, 1.3	0.13
Sex				-7.6	-26, 11	0.4	-8.9	-29, 11	0.4	-11	-31, 9.7	0.3
Occupation				6.8	0.59, 13	0.036	7.8	1.2, 14	0.025	9.4	2.6, 16	0.009
Processing Speed							0.05	-1.0, 1.1	>0.9	0.11	-0.95, 1.2	0.8
Attention							0.20	-0.42, 0.82	0.5	0.18	-0.45, 0.80	0.6
Working Memory							0.22	-1.0, 1.4	0.7	0.18	-1.0, 1.4	0.8
Learning							-0.31	-1.5, 0.84	0.6	-0.20	-1.4, 0.94	0.7
Inhibition							2.4	-1.1, 5.9	0.2	2.4	-1.1, 5.8	0.2
AQ										-1.0	-2.5, 0.46	0.2
ASRS - Attention										0.59	-1.3, 2.5	0.5
ASRS - Hyperactivity										0.87	-0.71, 2.5	0.3

Table S3. Linear regression analysis with four models adjusting for potential cofounders using Days of Sick Leave as dependent variable to study its relation to DF. Model 1 is without adjustments. In subsequent model adjustments for demographics and occupation (Model 2), cognitive functions (Model 3) and psychiatric traits (Model 4) have been added stepwise.

Table S4.

	Model 1			Model 2			Model 3			Model 4		
**Variable**	Beta	95% CI1	p-value	Beta	95% CI1	p-value	Beta	95% CI1	p-value	Beta	95% CI1	p-value
VF	-2.6	-4.9, -0.41	0.023	-2.4	-4.7, -0.10	0.045	-3.4	-6.1, -0.73	0.015	-4.1	-6.7, -1.4	0.004
Age				0.55	-0.08, 1.2	0.093	0.56	-0.12, 1.2	0.11	0.78	0.09, 1.5	0.031
Sex				-6.7	-26, 12	0.5	-11	-31, 9.4	0.3	-16	-36, 5.1	0.14
Occupation				6.6	0.20, 13	0.047	8.1	1.4, 15	0.020	9.8	3.2, 16	0.005
Processing Speed							-0.10	-1.2, 0.97	0.9	0.01	-1.0, 1.0	>0.9
Attention							0.07	-0.56, 0.69	0.8	0.05	-0.56, 0.66	0.9
Working Memory							0.43	-0.79, 1.6	0.5	0.35	-0.83, 1.5	0.6
Learning							-0.04	-1.2, 1.2	>0.9	0.17	-1.0, 1.3	0.8
Inhibition							3.3	-0.43, 6.9	0.088	3.6	-0.02, 7.2	0.056
AQ										-1.2	-2.6, 0.28	0.12
ASRS - Attention										1.2	-0.69, 3.1	0.2
ASRS - Hyperactivity										0.93	-0.62, 2.5	0.2

Table S3. Linear regression analysis with four models adjusting for potential cofounders using VF as dependent variable to study its relation to Days of Sick Leave. Model 1 is without adjustments. In subsequent models adjustments for demographics and occupation (Model 2), cognitive functions (Model 3) and psychiatric traits (Model 4) have been added stepwise.

Table S5.

	**DF1**	**DF2**	**DF3**
Pearson's r / p-value	-0.135 / 0.253	-0.291 / 0.012	-0.223 / 0.056
	**VF1**	**VF2**	**VF3**
Pearson's r / p-value	-0.277 / 0.017	-0.177 / 0.132	-0.233 / 0.046

Table S4. The independent contribution of DF1-3 and VF1-3 to sick leave in subjects who had at least one day of sick leave (n=74). Sick Leave log10 was used as outcome variable due to non-normal distribution in raw data.

References Supporting Information

Abe, C., Rolstad, S., Petrovic, P., Ekman, C.J., Sparding, T., Ingvar, M., and Landen, M. (2018). Bipolar disorder type I and II show distinct relationships between cortical thickness and executive function. *Acta Psychiatr Scand* 138, 325-335.

Baron-Cohen, S., Wheelwright, S., Skinner, R., Martin, J., and Clubley, E. (2001). The autism-spectrum quotient (AQ): evidence from Asperger syndrome/high-functioning autism, males and females, scientists and mathematicians. *J Autism Dev Disord* 31, 5-17.

Cieslik, E.C., Mueller, V.I., Eickhoff, C.R., Langner, R., and Eickhoff, S.B. (2015). Three key regions for supervisory attentional control: evidence from neuroimaging meta-analyses. *Neurosci Biobehav Rev* 48, 22-34.

Collie, A., Maruff, P., Makdissi, M., Mccrory, P., Mcstephen, M., and Darby, D. (2003). CogSport: reliability and correlation with conventional cognitive tests used in postconcussion medical evaluations. *Clin J Sport Med* 13, 28-32.

Delis, D.C., Kaplan, E., and Kramer, J.H. (2001). *Delis-Kaplan Executive Function System (D-KEFS) examiner's manual.*

San Antonio, Texas, USA: The Psychological Corporation. San Antonio, Texas, USA: The Psychological Corporation.

Delis, D.C., Kramer, J.H., Kaplan, E., and Holdnack, J. (2004). Reliability and validity of the Delis-Kaplan Executive Function System: an update. *J Int Neuropsychol Soc* 10, 301-303.

Diamond, A. (2013). Executive functions. *Annu Rev Psychol* 64, 135-168.

Furey, R.T., Bowden, S.C., Jewsbury, P.A., Sudarshan, N.J., and Connolly, M.L. (2024). Investigating the Latent Structure of Executive Function in the Delis-Kaplan Executive Function System Using Cattell-Horn-Carroll Theory. *Assessment* 31, 363-376.

Homack, S., Lee, D., and Riccio, C.A. (2005). Test review: Delis-Kaplan executive function system. *J Clin Exp Neuropsychol* 27**,** 599-609.

Karr, J.E., Hofer, S.M., Iverson, G.L., and Garcia-Barrera, M.A. (2019). Examining the Latent Structure of the Delis-Kaplan Executive Function System. *Arch Clin Neuropsychol* 34**,** 381-394.

Kessler, R.C., Adler, L., Ames, M., Demler, O., Faraone, S., Hiripi, E., Howes, M.J., Jin, R., Secnik, K., Spencer, T., Ustun, T.B., and Walters, E.E. (2005). The World Health Organization Adult ADHD Self-Report Scale (ASRS): a short screening scale for use in the general population. *Psychol Med* 35**,** 245-256.

Mace, R.A., Waters, A.B., Sawyer, K.S., Turrisi, T., and Gansler, D.A. (2019). Components of executive function model regional prefrontal volumes. *Neuropsychology* 33**,** 1007-1019.

Miyake, A., Friedman, N.P., Emerson, M.J., Witzki, A.H., Howerter, A., and Wager, T.D. (2000). The unity and diversity of executive functions and their contributions to complex "Frontal Lobe" tasks: a latent variable analysis. *Cogn Psychol* 41**,** 49-100.

Sakamoto, S., Takeuchi, H., Ihara, N., Ligao, B., and Suzukawa, K. (2018). Possible requirement of executive functions for high performance in soccer. *PLoS One* 13**,** e0201871.

Straume-Naesheim, T.M., Andersen, T.E., and Bahr, R. (2005). Reproducibility of computer based neuropsychological testing among Norwegian elite football players. *Br J Sports Med* 39 Suppl 1**,** i64-69.

Suchy, Y., Kraybill, M.L., and Gidley Larson, J.C. (2010). Understanding design fluency: motor and executive contributions. *J Int Neuropsychol Soc* 16**,** 26-37.

Vestberg, T., Reinebo, G., Maurex, L., Ingvar, M., and Petrovic, P. (2017). Core executive functions are associated with success in young elite soccer players. *PLoS One* 12**,** e0170845.
